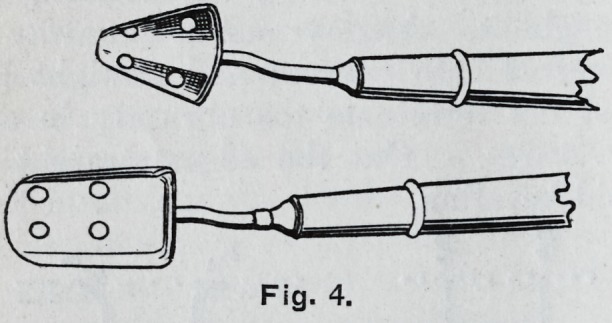# The Manipulation of Metallic Shell Inlays

**Published:** 1904-06

**Authors:** T. C. Trigger

**Affiliations:** St. Thomas, Ontario.


THE AMERICAN

JOURNAL OF DENTAL SCIENCE

### THE MANIPULATION OF METALLIC SHELL INLAYS.

By T. C. TRIGGER, D. D. S., St. Thomas, Ontario.

(Written for The American Journal of Dental Science.)

The restoration of lost portions of teeth by means of inlays is as old as the art of dentistry itself, but as new adaptations of old processes are constantly appearing, I need make no apology in discussing the subject as it has been presented to me in a practical way.

Gold inlays have been frequently used in very large cavities of the bicuspids and molars, the walls of which were too frail to support ordinary gold fillings, although they are applicable to almost any cavity except very shallow ones which will not permit of sufficient anchorage.

In preparing a cavity for an inlay, there are several requisites:

1. The cavity edges should be thoroughly prepared.

2. The cavity must possess such depth as will admit of good anchorage.

3. The matrix must be easily adapted to the margins of the cavity.

4. Thorough access must be had for its insertion.

5. There must be good retention.

In commencing the operation for inlay work, first cleanse the cavity of all decay, then proceed to trim the edges. In cutting the margins remove any overhanging portions, thereby making a uniform line throughout. Angles must be cut away and converted into curves; and straight lines must not terminate too abruptly in short curvatures. Cut the edges straight and avoid beveling.

Fig. 1.

Fig. 2.

After the edges have been thoroughly prepared construct a core for the adaptation of the matrix. Take cement or compound and insert it into the base of the cavity, filling it up nearly to the margin and across the whole floor of the cavity. By doing this it will prevent the unnecessary extension of the matrix to the base of the cavity, and at the same time minimize the quantity of gold used for the contouring.

Fig. 3 shows the core in position complete for the swedging of the matrix. In order to obtain an exact impression of the cavity so prepared, take a small piece of impression compound and force it into position; trim to the edge of the cavity and after it cools sufficiently, gently remove.

3 4 5 6

Fig. 3.

Having constructed several forms of trays suitable to convey the compound in position, this method may also be used to withdraw the matrix. Then from this run a cast to obtain a die and counter-die.

Fig. 4.

Pure gold is used for the matrix, and this can be had from supply houses in sheet form. But as this plate is too thick it should be passed through the mill, until the required thinness is obtained. Old gold fillings melted and rolled will answer. Cut a piece of gold somewhat larger than the die, place it in position, apply the counter-die and swage.

After having done this approximately shape the matrix, when it is ready to be further swaged, in the already prepared cavity. To obtain this end, it is compressed in position by various shaped rubbers, consisting of rings, cones, blocks and points which can be forced when required by blunt instruments, or they can each be attached to the end of points specially constructed to retain them. Dr. Walter M. Bruck in his book on "The Fillin[illegible] of Teeth with Porcelain," gives an illustration of instruments mounted for this purpose.

Many times without making a die, the matrix can be pressed and burnished in the cavity as described. Fig. 4 represents the matrix accurately in position and edges trimmed. It is advisable in large compound cavities in molars, involving the buccal and dorsal surfaces, to make the matrix in two pieces. By doing this it will prevent the buckling of the gold, and thus it is easier to correctly manipulate it in its adaptation to the cavity. When one has satisfied himself that the matrix is accurately adapted, gently remove by teasing it out of the cavity with a fine pointed instrument; then it is taken to the laboratory for further attention..

Take a stick of compound, soften one end, and then press gently into the external surface of the matrix. Care must be observed in doing this, as any undue pressure may change the form of the matrix.

The matrix being imbedded in the compound, drill holes for the insertion of pins. The pins should have well formed heads, and their length must be determined by the depth of the cavity. In vital teeth where the cavities are of limited depth insufficient for any extended attachment, countersunk holes should be used. To obtain this end, drill the number of holes desirable and insert points, made from lead from a lead-pencil. Penetrate them beyond the matrix, and after the filling in process they are removed, leaving holes so that the retentive material employed will insinuate itself in the openings thus left in the inlay.

At this stage, if the operator found it necessary he could vary his manipulation by any feasible method to further increase the retention of the inlay, such as by using both pins and countersunk depressions at the same time, or by using a small tube sufficiently long to obtain good anchorage at the base of the cavity and extending as high as the surface of the core. To retain this in position it would be well to solder at the lower end a small plate of metal, as will best suit the extent of the cavity. This tube attachment is cemented permanently in an upright position so that the wire anchorage, which is attached to the matrix, will accurately pass within the already cemented post which is in the core. The hollow post which is fastened in the tooth should extend slightly beyond the core, so that when in the act of compressing the matrix over it, it will leave a slight indention which will indicate where to penetrate for the anchor pin. It should fit into the hollow post accurately. (Fig. 5.)

Finally insert the matrix and all attachments in a mixture of plaster and sand to hold them in place, and now it is ready for the filling in process. Twenty-two caret gold should be used for soldering all the work throughout, as when completed it will give a decidedly rich appearance.

To cement the inlay in position select the very best and finest cement obtainable. After giving the cement sufficient time to set, the inlay must be trimmed, polished and burnished. Fig. 6.

Francis Mellersh of London, in his paper on the "Sealed Gold Inlay," describes a good method of anchoring the plug by running a deep retaining groove around the whole surface, but, in the one above recited there is an already existing space, for whatever attachment is necessary, and at the same time the greater the cement area—the greater the security of the inlay is assured.

It may be stated here that pure gold has the remarkable characteristic that causes cement to adhere to it in a most tenacious manner.

## Figures and Tables

**Fig. 1. f1:**
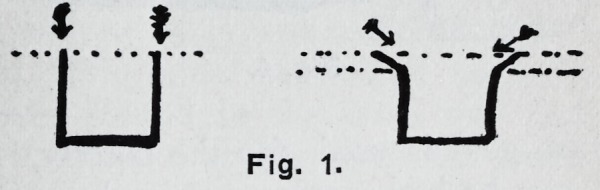


**Fig. 2. f2:**
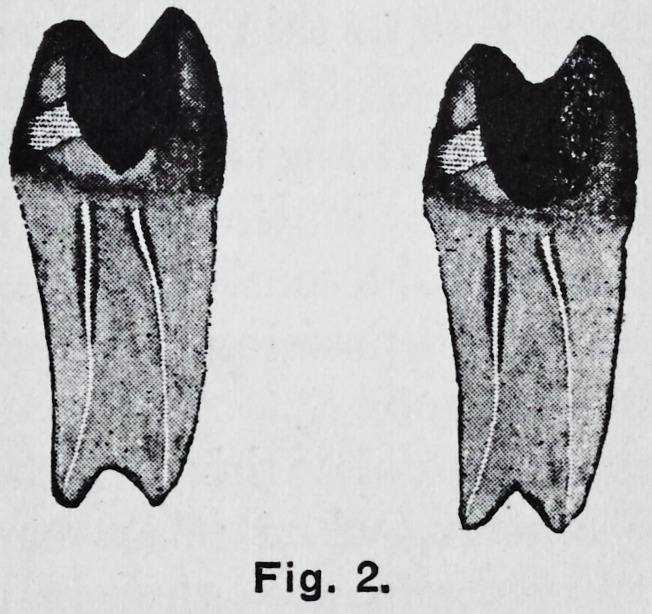


**Fig. 3. f3:**
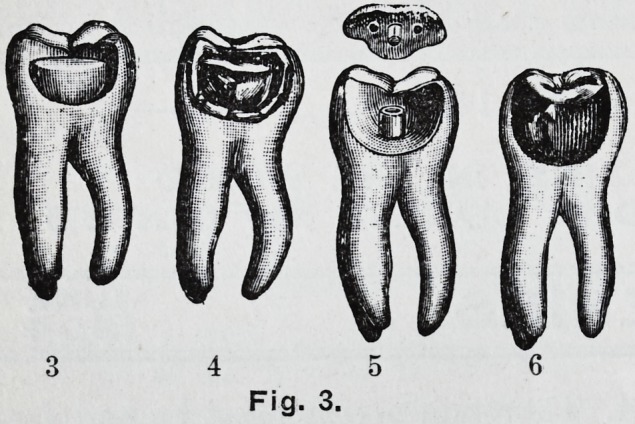


**Fig. 4. f4:**